# Transfer RNA-Derived Small RNAs in the Pathogenesis of Parasitic Protozoa

**DOI:** 10.3390/genes13020286

**Published:** 2022-01-31

**Authors:** Ruofan Peng, Herbert J. Santos, Tomoyoshi Nozaki

**Affiliations:** Department of Biomedical Chemistry, Graduate School of Medicine, The University of Tokyo, 7-3-1 Hongo, Bunkyo-ku, Tokyo 113-0033, Japan; ruofan@g.ecc.u-tokyo.ac.jp (R.P.); hjsantos@m.u-tokyo.ac.jp (H.J.S.)

**Keywords:** tRNA, tsRNA, tRNA-derived fragments, tRNA halves, protozoan parasites, extracellular vesicles

## Abstract

Transfer RNA (tRNA)-derived small RNAs (tsRNAs) are newly identified non-coding small RNAs that have recently attracted attention due to their functional significance in both prokaryotes and eukaryotes. tsRNAs originated from the cleavage of precursor or mature tRNAs by specific nucleases. According to the start and end sites, tsRNAs can be broadly divided into tRNA halves (31–40 nucleotides) and tRNA-derived fragments (tRFs, 14–30 nucleotides). tsRNAs have been reported in multiple organisms to be involved in gene expression regulation, protein synthesis, and signal transduction. As a novel regulator, tsRNAs have also been identified in various protozoan parasites. The conserved biogenesis of tsRNAs in early-branching eukaryotes strongly suggests the universality of this machinery, which requires future research on their shared and potentially disparate biological functions. Here, we reviewed the recent studies of tsRNAs in several representative protozoan parasites including their biogenesis and the roles in parasite biology and intercellular communication. Furthermore, we discussed the remaining questions and potential future works for tsRNAs in this group of organisms.

## 1. Introduction

Transfer RNAs (tRNAs) are essential adaptor molecules used to translate the information from nucleic acid to peptides and are highly conserved in all three domains of life. tRNAs can be distinguished from other RNAs by their unique characteristics, namely: (1) tRNAs have short sequences of 70–100 nucleotides, and are folded into a highly conserved cloverleaf secondary structure and an L-shaped tertiary structure [1], and (2) tRNAs are heavily modified at up to 20% of their nucleotides [2], which can prevent tRNA degradation [3] and affect amino acid charging [4,5] and protein biosynthesis [6]. tRNAs undergo several steps of posttranscriptional processing of the precursor tRNA transcript. From the initial transcripts transcribed by RNA polymerase III, precursor tRNA maturation involves the removal of the 5′ leader by RNase P, trimming of the 3′ trailer by RNase Z, addition of CCA by tRNA nucleotidyl transferase, splicing of intron(s) (if present), and multiple modifications of nucleoside residues [7]. The final product of mature tRNAs contains, from 5′ to 3′, the D loop, anticodon loop, variable loop, T loop, and the acceptor stem in their cloverleaf secondary structure.

tRNAs were first thought to be stable and, thus, small RNAs produced from tRNAs with a similar size as microRNAs (miRNAs) have been neglected. However, recent developments in unbiased high-throughput sequencing facilitated the discovery of a new class of non-coding RNA (ncRNA), tRNA-derived small RNAs (tsRNAs), which map to the known tRNA genes [8,9,10]. On the other hand, tRNA restriction systems that process tRNAs by digesting them into fragments have been discovered in both prokaryotes and eukaryotes [11,12,13,14]. The evidence suggests the role of tRNAs is far more than what we expected before, wherein tRNAs only participate in translation. The noncanonical role of tRNAs in prokaryotes [15] and mammals [16] have been well summarized. In this review, we focused on the tsRNAs in protozoan parasites, and their biological functions and contribution to parasitism.

## 2. tRNA-Derived Small RNAs (tsRNAs) in Protozoan Parasites

tsRNAs can be broadly categorized into tRNA halves and tRNA-derived fragments (tRFs). tRNA halves originate from a single cleavage at the anticodon loop of mature tRNAs and this process was first demonstrated under cellular stress condition catalyzed by angiogenin (ANG) [17]. They can be further classified as either 5′ tRNA halves or 3′ tRNA halves with a length range of 31 to 40 nucleotides [16]. tRFs can be generated by the endonucleolytic cleavage of both precursor and mature tRNAs in their D, anticodon, and T loops respectively, and are usually 14 to 30 nucleotides long [18]. According to the start and end positions, tRFs can be categorized into: (a) tRF-5 derived from 5′ end cleavage in the D loop of mature tRNAs, (b) tRF-3 generated from 3′ end of mature tRNAs cleaved in the T loop, (c) tRF-1 formed from the 3′ end containing poly-U sequence of precursor tRNA [9], and (d) internal tRFs (previously termed as tRF-2) arising from the anticodon loop of mature tRNAs [7]. Additionally, at least two other classes of tRFs, (e) tRNA intronic circular RNA (tricRNA) synthesized from intron-containing tRNAs during intron splicing [19] and (f) 5′ external transcribed spacer (ETS) and internal transcribed spacer (ITS) specifically present in prokaryotic precursor tRNAs [15,20] (will not be discussed here), have been reported. Due to the size and folding similarity of tsRNAs and miRNA, the endoribonuclease, Dicer, which is required for miRNA generation, has been implied to be associated with the formation of tRF-5/3 [8,21,22], though Dicer knockout cells showed no difference with wildtype cells in tRFs abundance [21,23,24].

**Figure 1:**
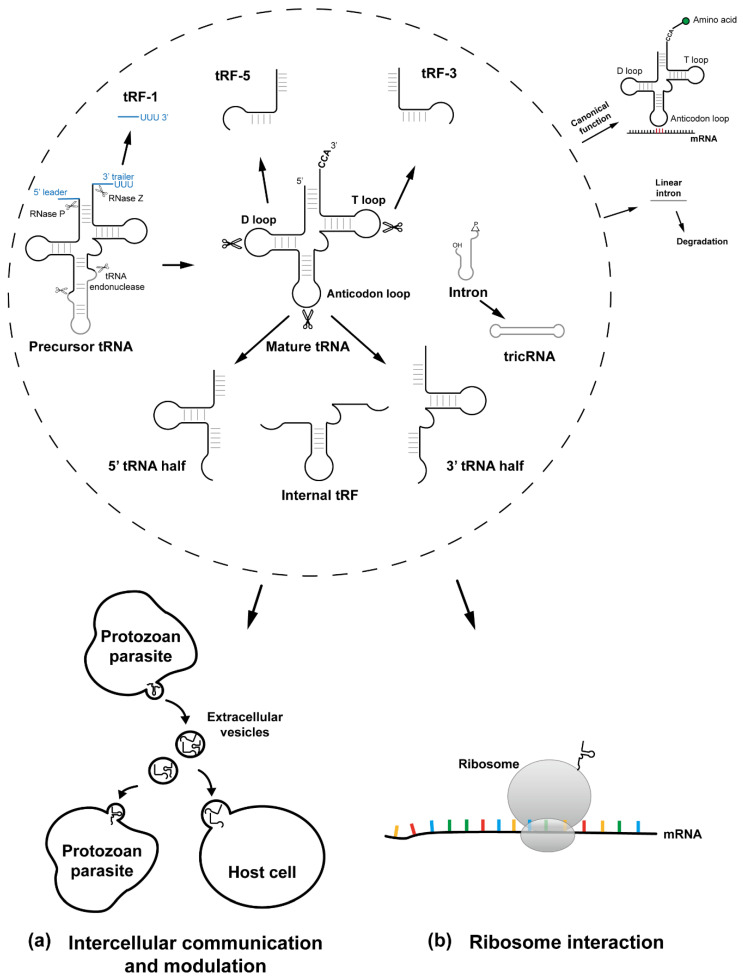
tRNA maturation, tRNA-derived small RNAs processing, and roles of tRNA-derived RNAs in protozoan parasites. Precursor tRNA from initial transcripts requires a series of post-transcriptional processing including the removal of the 5′ leader by RNase P, the trimming of the 3′ trailer by RNase Z, the addition of CCA by tRNA nucleotidyl transferase, the splicing of intron(s) (if present), and multiple modifications of nucleoside residues to form the mature tRNAs. Except for their canonical role as an adaptor during protein synthesis, mature tRNAs can be further processed to tRNA-derived RNAs after undergoing specific cleavage reactions. The species of tsRNAs can be categorized by the cleavage site, from 5′ to 3′, including: tRF-5, 5′-tRNA half, internal tRFs, 3′ tRNA half, and tRF-3. In intron-containing tRNAs, the intron can be circularized by a ligase specific for ligation of 2’,3′-cyclic phosphate cleavage site and perform as tRNAs, while the linear intron will be subsequently degraded. The biological functions of tsRNAs in representative protozoan parasites have been linked to the regulation of cellular differentiation, stress response, and parasite-parasite or parasite-host interaction by fundamental mechanisms of: (**a**) tsRNAs export via extracellular vesicles to communicate to other parasites or to host cells leading to a variety of downstream phenotypic changes, (**b**) tsRNAs interact with ribosome and affect protein synthesis reported in *Trypanosoma brucei*.

As mentioned, in some mammalian cells, tRNA halves were found to be produced by tRNA cleavage of specific nucleases such as Dicer and ANG under stressed conditions [17,25,26]. Nevertheless, tsRNAs are also present in non-stressed conditions [27], and can even be packed into extracellular vesicles (EVs) [28,29,30,31,32,33]. Recent studies on tsRNAs in protozoan parasites have highlighted the significance of the roles of tsRNAs in parasite biology, pathogenesis, and parasite-host interaction. The biogenesis of tsRNAs in eukaryotes and the biological functions reported in representative protozoan parasites are depicted in Figure 1. Also, a summary of tsRNA abundance and major findings among these organisms are shown in Table 1.

### 2.1. Trypanosoma cruzi and Trypanosoma brucei

*Trypanosoma cruzi* and *T. brucei* are two species of dixenous (or heteroxenous) kinetoplastid parasites that cause diseases in mammals including humans. *T. cruzi,* which causes American trypanosomiasis, also known as Chagas’ disease in humans, is endemic in Latin America [34]. It was firstly reported that about 25% of small RNAs sequenced in *T. cruzi* were assigned to the tRNA category and are derived mainly from tRNA^Asp^(GUC), tRNA^Glu^(CUC), and tRNA^Ala^(CGC) as tRNA halves [35] (Table 1). The production of these tRNA halves could be significantly induced by nutritional stress [35].

It has been reported that the invasion efficiency of *T. cruzi* trypomastigotes (infective stage) increased when purified parasite EVs were administered to mice prior to infection. Aside from tissue parasitism, EVs also enhanced inflammation via production of interleukin (IL)-4 and IL-10 in affected mice, suggesting that EVs play a crucial role in *T. cruzi* pathogenesis [36]. In another report, purified *T. cruzi* EVs that were coincubated with HeLa cells caused modifications in the host-cell cytoskeleton, extracellular matrix, and immune response pathways [37]. Interestingly, enriched tsRNAs were also identified in the *T. cruzi* EVs. When specific tsRNAs isolated from EVs were introduced into HeLa cells, they elicited similar responses to when HeLa cells were incubated with EVs [37]. HeLa cells transfected with tsRNA^Thr^ demonstrated modified expression of genes related to host parasite interactions, including three genes encoding activating transcription factor 3 (ATF3), chemokine (C-X-C motif) ligand 2 (CXCL2), and dual specificity phosphatase 6 (DUSP6) respectively. ATF3 and CXCL2 are involved in immune response regulation, while DUSP6 is involved in a signaling cascade that affects adhesion, growth, proliferation, cytoskeleton regulation, and survival pathways [37]. Interestingly, in silico analysis identified putative tsRNA^Thr^ binding sites in ATF3, CXCL2, and DUSP6, suggesting that these genes are direct targets of tsRNA^Thr^, although experimental confirmation has not been conducted to ascertain the mechanism of modified expression of the aforementioned genes [37]. Also, EVs collected from stressed epimastigotes, which is the life cycle stage in the insect’s midgut, contained small RNAs derived from rRNA and tRNA. The majority (>90%) of the tRNA fragments were derived from tRNA^Leu^, tRNA^Thr^, tRNA^Glu^, tRNA^Gly^, and tRNA^Arg^, respectively [38]. Overall, the small RNA content, including tRNA-derived fragments, of *T. cruzi* EVs varies between parasite stages: non-infective epimastigotes and infective metacyclic trypomastigotes [39]. It is plausible that this differential expression and composition of EV small RNAs play regulatory functions once the EVs are transferred from parasite to parasite as well as from parasite to mammalian host cells, although studies that elucidate molecular mechanisms have not been reported.

Another kinetoplastid, *Trypanosoma brucei*, is the etiologic agent of African trypanosomiasis or sleeping sickness, endemic in sub-Saharan Africa [40]. In contrast to *T. cruzi*, *T. brucei* is an extracellular parasite and undergoes cell division in the host bloodstream and the vector’s gut and salivary glands [41]. It is transmitted by the injection of infectious metacyclic trypomastigotes to the host’s skin through the bite of its vector, the tsetse fly [42]. So far, it has been known that *T. brucei* utilizes ribosome-associated non-coding RNAs (rancRNAs) in regulating translation in response to various forms of stress [43]. One particular molecule that has been identified to interact with ribosomes and polysomes and is significantly increased during starved conditions and during the stationary phase in the procyclic form (the life cycle stage in the tsetse midgut) is the 3′ tRNA^Thr^ halves [43]. Additionally, all nutrient-deprivation induced 3′ tRNA^Thr^ halves lack the 3′ CCA, which is likely produced from mature tRNAs lacking the 3′ CCA while being cleaved. Ribosome interaction and enhanced protein biosynthesis by 3′ tRNA^Thr^ halves was demonstrated both in vitro and in vivo, as one of the parasite’s mechanisms of stress recovery during starvation [43].

### 2.2. Leishmania donovani and Leishmania braziliensis

Species belonging to the genus *Leishmania* cause leishmaniasis, a disease that is highly endemic in tropical and subtropical nations. Infection is transmitted by sandfly vectors that carry flagellated promastigotes upon blood meal to mammals including rodents, dogs, and humans. Host monocytes in the bloodstream phagocytose promastigotes and are trafficked into phagolysosomes where the parasites differentiate into amastigotes followed by active cell division [53]. Infection with *L. donovani* causes visceral leishmaniasis, a disease that can be fatal if left untreated. Whereas *L. braziliensis* infection leads to a milder disease, mucocutaneous leishmaniasis, it may lead to debilitating effects to affected individuals by disfigurement of critical soft tissue structures [44].

During infection, *Leishmania* releases exosomes that contain a number of proteins which have previously been characterized as molecules that elicit stronger pathogenicity of the parasite [54]. In addition, *Leishmania* exosomes also pack small non-coding RNAs (ncRNAs) which originated mostly from tRNAs and rRNAs [44]. High-throughput sequencing of exosomal RNA derived from culture supernatant of axenic *L. braziliensis* and *L. donovani* amastigotes (the proliferative stage of the parasite) revealed a 36.4% and 21.1% of total reads, respectively, mapped to tRNA genes [44]. The fact that the sizes of a majority of the small ncRNA transcripts in both *L. donovani* and *L. brazilensis* are between 20 to 40 nucleotides and the mean length of reads mapped to tRNAs are 38 and 46 nucleotides, respectively, suggests the major presence of tRNA halves [44]. For both *Leishmania* species, tsRNAs derived from tRNA^Asp^, tRNA^Gln^, tRNA^Glu^, and tRNA^Leu^ were most abundantly present. Among these tsRNAs, the most abundant tsRNA^Asp^ and tsRNA^Gln^ represented 5′ tRNA halves (>50%), though 3′ tRNA halves and tRF-5/3 are also present as a minority [44]. Exosomal RNA cargoes were demonstrated to be delivered to macrophages in vitro; however, their role is still unknown. It has been previously postulated that intercellular communication mediated by exosome uptake may be linked to a yet-unrecognized mechanism of *Leishmania* pathogenesis [44].

### 2.3. Plasmodium falciparum

*Plasmodium falciparum* is one of five *Plasmodium* species that causes malaria in humans, affecting 241 million people worldwide and causing an estimated 627,000 deaths, with 68% of the additional deaths attributed to service disruptions caused by the ongoing COVID-19 pandemic [55]. Upon blood meal, *Anopheles* mosquitos introduce sporozoites to humans [56]. From the skin, these sporozoites travel through the bloodstream, reaching the liver and invading hepatocytes, where they differentiate into merozoites. Multiple replication rounds produce tens of thousands of merozoites which are then released from parasitized hepatocytes back into the bloodstream where they spread and invade red blood cells (RBCs) [57]. Invasion of erythrocytes by merozoites occur as the parasite latches itself on the erythrocyte plasma membrane, initially invaginating then sealing to form a parasitophorous vacuole, which is both a secondary barrier for the parasite as well as a hub for communication and exchange with the host [58]. Indeed, malaria-infected red blood cells (iRBCs) have been demonstrated to quantitatively release EVs, particularly microvesicles, which can be transferred between parasites, enabling them to regulate formation of transmission stages [59].

MicroRNAs (miRNAs) were found to be absent in *P. falciparum* and other *Plasmodium* species [60]. The lack of Dicer and Argonaute (AGO) homologs, which are responsible for miRNA cleavage, suggests that *P. falciparum* may utilize an alternative pathway for post-transcriptional regulation [45]. Despite this, recent findings are suggestive of *Plasmodium* having the capability to manipulate host miRNA production as seen from differential miRNA profiling of plasma samples from patients with severe and uncomplicated malaria respectively [61,62].

In another study, small RNAs with sizes ranging from 18 to 30 nucleotides from *P. falciparum* blood stage parasites were deep-sequenced [45]. Analysis revealed 22.4% of small RNAs (18 to 30 nucleotides) were originated from tRNA-coding genes [45]. Due to the limitation of small RNAs size-fractionation at 18 to 30 nucleotides, only tRFs but not tRNA halves were focused on in this previous survey [45]. tRFs in *P. falciparum* are mainly derived from tRNA^Pro^, tRNA^Phe^, tRNA^Asn^, tRNA^Gly^, tRNA^Cys^, tRNA^Gln^, tRNA^His^, and tRNA^Ala^. These tRFs are predominated by tRF-5 (86.2%), while internal tRFs and tRF-3 only occupy for 6.2% and 6.0%, respectively [45]. EVs isolated from iRBCs have also been analyzed for their small RNA composition and results revealed that the most abundant tRNA, tRNA^Gly^, was derived from 5′ and 3′ tRNA halves [46]. Furthermore, the analysis revealed 5′ end tRNA halves of mostly 30 to 34 nucleotides to have originated from tRNA^Lys^ and tRNA^Phe^ as specific cleavage products and their roles have been posited to be connected to intercellular communication as suggested by the confirmation of RNA transfer to human bone marrow endothelial cells [46].

### 2.4. Toxoplasma gondii

Toxoplasmosis is a disease caused by the coccidian parasite *Toxoplasma gondii*. Although usually self-limiting, serious complications may manifest among the immunocompromised or the immunosuppressed, as well as during pregnancy. The complex life cycle of *T. gondii* involves the sexual developmental stages in cats and the asexual stages in a range of intermediate hosts. Transmission in feline hosts occurs during ingestion of tissue cysts present in infected prey. On the other hand, transmission to humans may be through the ingestion of either infectious oocysts from cat feces, or tissue cysts or tachyzoites present in raw or undercooked meat, and by acquisition of tachyzoites found in contaminated blood or tissue transplants, or unpasteurized milk [63].

Investigation of small RNAome (20 to 40 nucleotides) of *Toxoplasma gondii* by high-throughput deep sequencing has been conducted, revealing that up to 34.78% of readout repertoire represents tRNA products [64]. Upon sequencing of 100 clones from size-fractionated cDNA libraries (20 to 50 nucleotides) extracted from extracellular *T. gondii* tachyzoites of the PLK strain (genotype II), 10 different species consisting of either 5′ or 3′ tRNA halves cleaved in the anticodon loop were determined by northern blot [47]. However, the loss of CCA in the sequence of these 3′ tRNA halves raise the question about whether tRNA halves in *T. gondii* are generated before maturation of precursor tRNAs or from the mature tRNAs whose CCA was removed [47]. The existence of the first cytosine of the CCA sequence in 3′ tRNA half of tRNA^Tyr^(GUA) and the conserved enzymes of the tRNase Z family in *T. gondii*, which was reported in *Escherichia coli* to cleave off the CCA sequence from mature tRNAs [65], points to the suggestion that tRNA halves formation in *T. gondii* may happen in mature tRNAs whose 3′ CCA had been removed prior to the cleavage of their anticodon loop [47]. The evidence of higher production of tRNA halves in avirulent strains and in metabolically quiescent stages shed light on the function of tRNA halves in *T. gondii* to down-regulate basal metabolism at specific stages of parasite development [47].

### 2.5. Entamoeba histolytica

It has been demonstrated that tRNA splicing and 2’, 3′-cyclic phosphate ligases are conserved in the amoebozoan *Entamoeba histolytica*, suggesting the possibility of the presence of tRNA intronic circular RNAs [66]. This anaerobic protozoan parasite is the causative agent of amebiasis in humans, a disease often characterized by diarrhea, but could lead to more severe symptoms such as abscess formation upon invasion of the lungs, liver, or brain [67]. Amebiasis is transmitted by the ingestion of cysts from fecally-contaminated food and/or water. Cysts convert to trophozoites at the terminal ileum. Trophozoites actively reproduce and parasitize the large intestine by adhesion, protease secretion, phago/trogocytosis, invasion, and eventually undergo encystation within the colon before being released via excretion of feces by the host [68]. *E. histolytica* also secrete EVs whose proteome consists of an assortment of proteins involved in vesicle membrane production, molecular binding, signal transduction, and structural molecular activity [69]. Small RNAs were also detected in the EVs of *E. histolytica*, and sequence analysis identified selective sorting of a population of antisense small RNAs of ~27 nucleotides [69]. Similar to the role of EVs in other organisms, amoebic EVs are utilized for intercellular communication. This was clearly demonstrated by the promotion of encystation when EVs isolated from encysting parasites were added to trophozoites, and conversely by the inhibition of encystation when EVs collected from active trophozoites were introduced to encysting cells [69].

It has also been shown that amoebic EVs contain tRNA halves and tRFs which associate with *E. histolytica* AGO proteins *Eh*AGO2-2 and *Eh*AGO2-3 [48]. Prior to EV collection, parasites were cultivated in serum-free medium to prevent contamination with bovine serum EVs. Thus, serum starvation is a trigger that prompted increased production of tRNA halves which is common in other organisms where tRNA cleavage into halves is induced during nutritional and oxidative stress [17,70,71,72,73]. Most of the tRNA halves originated from a few tRNAs, particularly tRNA^Ala^(AGC), tRNA^Ala^(UGC), tRNA^Arg^(UCU), and tRNA^Asp^(GUC) [48]. The biogenesis of tsRNA can be highly affected by tRNA modifications [16]. It has also been reported that queuine can induce a C38 hypermethylation in the tRNA^Asp^(GUC) anticodon loop to promote the resistance of the parasite against oxidative stress [74], thus suggesting that a modulating mechanism may exist to regulate the synthesis of tRNA halves in this organism. However, the clear role of tRNA halves in the EVs of *E. histolytica* remains unknown, although it is hypothesized that they mediate intercellular communication [48].

### 2.6. Trichomonas vaginalis

The parabasalian parasite *Trichomonas vaginalis* causes trichomoniasis, one of the most common sexually transmitted infections in the world. It resides in the urogenital tract, causing vaginitis or urethritis in females and males respectively. This parasite induces inflammation and disruption of the host microbiota, and infection contributes to increased acquisition and transmission of human immunodeficiency virus [75]. In a global survey of small RNAs (18 to 40 nucleotides) in *T. vaginalis*, microRNAs were suggested to be absent in this organism, while 12.79% of clean reads were accounted as tsRNAs [49]. These tsRNAs were found to have originated from tRNA^Glu^, tRNA^Gly^, tRNA^Phe^, tRNA^Lys^, tRNA^Val^, tRNA^Arg^, tRNA^Asn^, and tRNA^Tyr^. All tRF-5/3 and 5′/3′ tRNA halves could be classified from these global tsRNAs in *T. vaginalis* [49]. The parasite mainly exists as a highly motile and infective trophozoite form, which may differentiate to the pseudocyst form whose pathology and role in the life cycle has not yet been clarified [76]. In *T. vaginalis*, snRNAs such as tRNA-halves and tRFs may be utilized during cellular differentiation as parasites undergo morphological changes, DNA replication, nuclear division, transposon activities, and lateral gene transfer [49]. Furthermore, *T. vaginalis* is also known to release EVs which are utilized to mediate communication across different parasite strains, which have led to enhanced adherence to human cervical epithelial cells [77], as well as with the host cells, which prompted an immunomodulatory effect by promoting IL-10 and inhibiting IL-6, IL-8, IL-13, and IL-17 production respectively [77,78]. EVs from this parasite were also demonstrated to contain mostly tRFs (88.2%) with lengths varying from 17 to 51 nucleotides after deep sequencing of the total small RNAs in the EV fraction [50]. Nine fragments with unique sequences were identified as tRFs, and all of them were derived from 5′ tRNAs whose lengths vary between 24 to 36 nucleotides, including seven 5′ tRNA halves and two tRF-5 [50]. So far, host uptake and subsequent downstream effects brought about by this class of RNAs contained in the EVs of *T. vaginalis* have not yet been demonstrated experimentally.

### 2.7. Giardia lamblia

*Giardia lamblia* is a unicellular protozoan parasite that causes giardiasis, one of the most common infectious human diseases around the world [79]. Its life cycle involves two stages: the vegetative and the infectious stage, featuring differentiation from trophozoite to cyst (encystation) and vice versa (excystation). A sequencing analysis conducted on the total RNA of *G. lamblia* trophozoites and encysting cultures revealed a ~50 nucleotide encystation-specific band. Further analysis showed that these ~50 nucleotide tsRNAs were derived mostly from 3′ tRNAs that lack 3′ CCA, with a length of 44 to 49 nucleotides due to the cleavage sites at the anticodon left arm [51]. All the tsRNA were shown to be derived from tRNA^Glu^(CUC), tRNA^His^(GUG), and tRNA^Cys^(GCA). Additionally, formation of giardial tsRNAs could be induced by nutrient starvation and incubation at a low temperature, which suggests tsRNAs’ role of being regulators of protein synthesis under stressed and low metabolic conditions in this organism. Deep sequencing of small RNA of 18 to 40 nucleotides, tRF-5, tRF-3, and internal tRFs could also be involved in the differentiation process of *G. lamblia* [52]. Although the biogenesis of tsRNAs in *G. lamblia* has been proven, the lack of information of tsRNAs in EVs and other biological functions except for cellular differentiation remains an enigma that requires further investigation.

## 3. Conclusion and Future Perspectives

tRNA fragments were first described in *E. coli* in response to bacteriophage infection [80]. Furthermore, investigation of tsRNAs have mushroomed from prokaryotes to mammals, revealing that under stressed or non-stressed conditions, tsRNAs can perform as signaling molecules or gene expression regulators. It has been shown and reviewed that tsRNAs biogenesis is conserved in various parasites. These tsRNAs are involved in cellular differentiation and stress response to help the parasites tackle complex environments during infection of their hosts. Additionally, by releasing tsRNAs, parasites may be able to regulate gene expression in parasite populations or host cells to benefit their parasitic lifestyle. Additionally, by releasing tsRNAs, parasites may be able to regulate gene expression in parasite populations or host cells to benefit their parasitic lifestyle (Figure 1).

Recent developments in bioinformatics have provided several tRF databases for tsRNA research [81,82,83]. In mammals, the biogenesis and abundance of tsRNAs have been revealed. Functionally, they carry out important roles in several diseases including cancer [84,85,86,87] and metabolic/neurological disorders [88,89,90,91,92,93,94,95] by regulating protein expression via various mechanisms [16]. The manner of protein expression regulation mediated by tsRNAs include: (a) direct interaction with key molecules involved in translation [96,97,98], (b) coupling with AGO proteins to target base-pairing mRNA [21,25,99], which is similar to the mechanism facilitated by miRNAs, and (c) sequestration of RNA-binding proteins, and in effect, deprivation of other mRNAs or non-coding RNAs that require interaction with proteins for stability and homeostasis [85,100,101,102,103].

Compared with mammals, protozoan parasites deal with more complicated and dynamic environments during infection. Interestingly, a growing amount of evidence has implicated parasite tsRNAs to regulatory functions not only intracellularly but also intercellularly, which often contributes to their parasitism. The stress-induced synthesis of tRNA halves shown in mammals [96] could be consistently observed in several parasites as reviewed in this paper, suggesting a conserved mechanism of inducing tsRNA formation in both protozoans and metazoans. In stem cells, pseudouridine synthase 7 (PUS7) was observed to be essential for the pseudouridylation of tRF-5s, and the lack of PUS7 impaired the activation of tRF-5s and the corresponding tRF-mediated translation regulation, thus, finally, impairing differentiation [96]. Similar mechanisms may be implied in parasites, as one of the remarkable features of these organisms is their cell-stage dynamism in response to environmental stimuli. tsRNAs, specifically those that are released in EVs, appear to play a common role in stage conversion across different protozoan parasite species. tsRNA-containing EVs released by malaria-infected RBCs and *E. histolytica* were shown to guide the stage-transformation decision of parasite populations [59]. More interestingly, tsRNAs in EVs can contribute to intercellular communication among not only the same population but also across other species. In *T. cruzi,* tsRNAs released via EVs were suggested to initiate interaction with host cells, which supports parasitism [37]. A similar phenomenon was reported in *L. donovani*, and *L. braziliensis*; however the downstream effects of tsRNAs taken up by macrophages have not yet been reported [44].

As summarized in this review, tsRNAs have integral roles not only in the biology of representative parasitic protozoa, but also in implementing parasitism and pathogenesis. Packaging of tsRNAs into EVs proves to be an efficient, evolutionarily conserved process in eliciting appropriate responses from sender to recipient cells. It is expected that tsRNAs will be demonstrated in other protozoan parasites and that their roles, albeit similar to what has been reported, could also be lineage-specific. Although research on tsRNAs in protozoan parasites has recently been gaining momentum, a large knowledge gap on the detailed biological functions still exists in most of the other parasites. Additionally, technical issues such as heavy modifications on tRNA which have been known to hamper reverse transcription, and difficulty in analysis of smaller-size RNA must be overcome to get a bias-less pattern of tsRNAs in the future. Other major unanswered questions of tsRNAs in protozoan parasites include the identification of specific targets of parasite-released tsRNAs in either parasites or host cells, which can vary upon different tRNA isoacceptors and species, the global pattern changes of tsRNAs released upon different stages in the life cycle, which is usually complicated in parasites, and the characterization of the tsRNAs derived from precursor tRNAs (if present) including 5′ leader, 3′ trailer, and tRNA intronic circular RNAs, which are relatively neglected.

## Figures and Tables

**Table 1:** tsRNAs abundancy and major findings in protozoan parasites.

Protozoan Species	Category of Identified tsRNAs	Validated or Proposed Functions and Important Findings	Predominant tRNAs That tsRNAs Were Identified for	References
*Trypanosoma cruzi*	tRNA halves	Induced by nutritional stress	tRNA^Asp^(GUC), tRNA^Glu^(CUC), and tRNA^Ala^(CGC)	[35]
	tRNA halves	Enriched in extracellular vesicles and transferred to host cells. Secreted tRNA^Thr^ halves directly regulate gene expression in HeLa cells.	tRNA^Leu^, tRNA^Thr^, tRNA^Glu^, tRNA ^Gly^, and tRNA^Arg^	[37,38]
*Trypanosoma brucei*	tRNA halves	3′ tRNA^Thr^ halves stimulate translation for stress recovery during starvation.	tRNA^Thr^, tRNA^Ala^, and tRNA^Asp^	[43]
*Leishmania donovani* and *Leishmania braziliensis*	Mostly tRNA halves	Contained in exosomes. Exosomal RNA cargoes can be delivered to macrophages.	tRNA^Asp^, tRNA^Gln^, tRNA^Glu^, and tRNA^Leu^	[44]
*Plasmodium falciparum*	Mostly tRF-5	Unknown	tRNA^Pro^, tRNA^Phe^, tRNA^Asn^, tRNA^Gly^, tRNA^Cys^, tRNA^Gln^, tRNA^His^, and tRNA^Ala^	[45]
	Mosly tRNA halves	Exosomal RNA cargoes can be delivered to human endothelial cells.	tRNA^Glu^, tRNA^Ser^, tRNA^Met^, tRNA^Pro^, tRNA^Arg^, tRNA^Trp^, tRNA^Arg^, and tRNA^Cys^	[46]
*Toxoplasma gondii*	tRNA halves	Production of tRNA halves is higher in avirulent strains and in metabolically quiescent stages	tRNA^Ala^(UGC), tRNA^Gln^(CUG), tRNA^Gly^(GCC), tRNA^Pro^(UGG), tRNA^Met^(CAU), and tRNA^Gly^(UCC)	[47]
*Entamoeba histolytica*	tRNA halves and tRFs	Assumed to mediate intercellular communication.	tRNA^Ala^(AGC), tRNA^Ala^(UGC), tRNA^Arg^(UCU), tRNA^Asp^(GUC)	[48]
*Trichomonas vaginalis*	tRNA halves and tRFs	Unknown	tRNA^Glu^, tRNA^Gly^, tRNA^Phe^, tRNA^Lys^, tRNA^Val^, tRNA^Arg^, tRNA^Asn^, and tRNA^Tyr^	[49,50]
*Giardia lamblia*	tRNA halves and tRFs	Low metabolic conditions can induce formation of tsRNAs.	tRNA^Glu^(CUC), tRNA^His^(GUG), and tRNA^Cys^(GCA)	[51,52]
